# Glutaric acid–2-(pyridin-4-yl)-1*H*-benzimidazole (1/1)

**DOI:** 10.1107/S1600536811049695

**Published:** 2011-11-30

**Authors:** Songzhu Lin, Ruokun Jia, Aimin He, Xiaoli Gao

**Affiliations:** a Northeast Dianli University, Jilin 132012, People’s Republic of China

## Abstract

The crystal structure of the title co-crystal, C_12_H_9_N_3_·C_5_H_8_O_4_, N—H⋯O and O—H⋯N hydrogen bonds link the components. There are also π–π stacking inter­actions between the imidazole rings, between the imidazole and pyridine rings and between the pyridine and benzene rings [centroid–centroid distances = 3.643 (2), 3.573 (2) and 3.740 (1)Å, respectively].

## Related literature

For background to hydrogen bonds, see: Moorthy *et al.* (2002 ▶); Muthuraman *et al.* (2000 ▶); Nangia & Desiraju (1999 ▶); Bhattacharjya *et al.* (2004 ▶). For related structures, see: Bei *et al.* (2000 ▶); Ozbey *et al.* (1998 ▶).
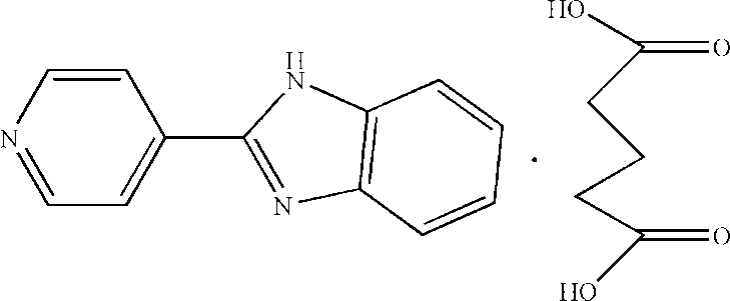


## Experimental

### 

#### Crystal data

C_12_H_9_N_3_·C_5_H_8_O_4_
*M* *_r_* = 327.34
Triclinic, 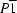

*a* = 7.4384 (15) Å
*b* = 8.9911 (18) Å
*c* = 11.868 (2) Å
α = 86.67 (3)°
β = 81.66 (3)°
γ = 85.57 (3)°
*V* = 782.1 (3) Å^3^
*Z* = 2
Mo *K*α radiation
μ = 0.10 mm^−1^
*T* = 293 K
0.20 × 0.17 × 0.15 mm

#### Data collection

Enraf–Nonius CAD-4 diffractometer
6041 measured reflections
2664 independent reflections
1657 reflections with *I* > 2σ(*I*)
*R*_int_ = 0.024
3 standard reflections every 100 reflections  intensity decay: none

#### Refinement

*R*[*F* ^2^ > 2σ(*F* ^2^)] = 0.040
*wR*(*F* ^2^) = 0.149
*S* = 1.11
2664 reflections
226 parameters
1 restraint
H atoms treated by a mixture of independent and constrained refinement
Δρ_max_ = 0.35 e Å^−3^
Δρ_min_ = −0.32 e Å^−3^

### 

Data collection: *CAD-4 Software* (Enraf–Nonius, 1989 ▶); cell refinement: *CAD-4 Software*; data reduction: *NRCVAX* (Gabe *et al.*, 1989 ▶); program(s) used to solve structure: *SHELXS97* (Sheldrick, 2008 ▶); program(s) used to refine structure: *SHELXL97* (Sheldrick, 2008 ▶); molecular graphics: *SHELXTL* (Sheldrick, 2008 ▶); software used to prepare material for publication: *WinGX* (Farrugia, 1999 ▶).

## Supplementary Material

Crystal structure: contains datablock(s) global, I. DOI: 10.1107/S1600536811049695/fj2466sup1.cif

Structure factors: contains datablock(s) I. DOI: 10.1107/S1600536811049695/fj2466Isup2.hkl

Supplementary material file. DOI: 10.1107/S1600536811049695/fj2466Isup3.cml

Additional supplementary materials:  crystallographic information; 3D view; checkCIF report

## Figures and Tables

**Table 1:** Hydrogen-bond geometry (Å, °)

*D*—H⋯*A*	*D*—H	H⋯*A*	*D*⋯*A*	*D*—H⋯*A*
N1—H1*A*⋯O2^i^	0.86	2.10	2.957 (3)	176
O1—H2⋯N3^ii^	0.87 (1)	1.75 (1)	2.615 (3)	173 (4)
O4—H1⋯N2	1.02 (4)	1.71 (4)	2.686 (3)	158 (3)

Symmetry codes: (i) 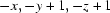; (ii) 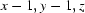.

**Fig. 1.**
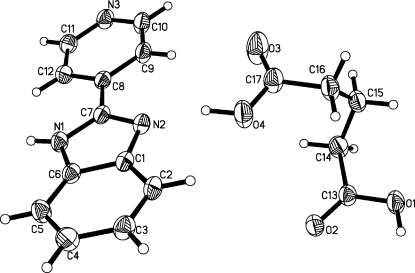
The structure of the title compound showing 30% probability displacement ellipsoids and the atom-numbering scheme.

**Fig. 2.**
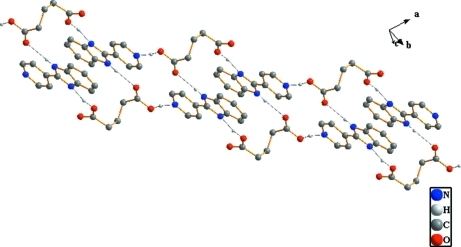
One-dimensional bend structure of the title compound.

**Fig. 3.**
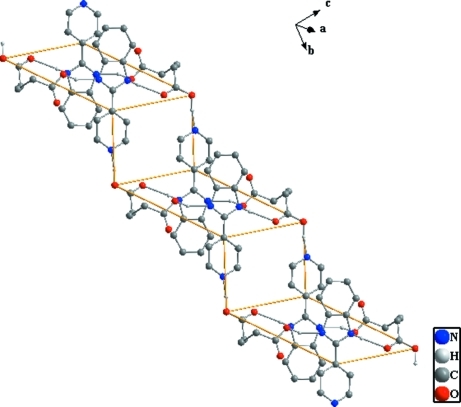
Two-dimensional structure of the title compound.

## supplementary crystallographic information

### Comment

The strong (O—H···O) and weak (C—H···O) hydrogen bonds, the halogen bond (C—*X*···O) and the weak C—H···π interaction, have been well characterized and exploited in the design of molecular assemblies (Moorthy *et al.*, 2002; Muthuraman *et al.*, 2000; Nangia and Desiraju, 1999; Bhattacharjya *et al.*, 2004). Our interest in benzimidazole stems from their biological activity (Bei *et al.*, 2000; Ozbey *et al.*, 1998). In this paper, we sysnthesized the title compound and report its structure.

Scheme I

The compound consists of 2-(pyridin-4-yl)-1*H*-benzimidazole and glutaric acid. In the title compound, the dihedral angle between the imidazole and the benzene was 1.40 (2)°, while the benzimidazole and the pyridine was 5.25 (1)°. It results that the all atoms in the 2-(pyridin-4-yl)-1*H*-benzimidazole are not coplanar strictly. In the part of glutaric acid, four atoms O1, O2, C13, C14 are lying in a same plane (p1) with the maximum diviation of 0.002° for C13, while other four atoms O3, O4, C16, C17 lying in another plane (p2) with the maximum diviation of 0.001% for O3. The dihedral angle between p1 and p2 is 10.50 (2)°.

In the lattice, there exist some kinds of hydrogen bonds. It forms one-dimension stairway structure between 2-(pyridin-4-yl)-1*H*-benzimidazole and glutaric acid *via* N—H···O, O—H···N hydrogen bonds (figure 2a and 2 b). Two adjacent strairway chains formed two dimension structure *via* the C—H···O intermolecular interaction.

In addition, there exists some π–π interactions between the rings [*Cg*1···*Cg*1=3.643 (2), *Cg*1···*Cg*2= 3.573 (2) and *Cg*2···*Cg*3=3.740 (1), respectively (*Cg*1, *Cg*2, *Cg*3 refer to the centroid of imidazole N1, C1, C6, N2, C7; the pyridine N3, C8, C9, C10, C11, C12 and the phenyl ring C1, C2, C3, C4, C5, C6, respectively)]. The π–π interaction, as well as the inter- and intra- hydrogen bond stabilized the crystal structure.

### Experimental

The title compound was obtained by 2-Pyridin-4-yl-1*H*-benzoimidazole (0.020 g, 0.1 mmol) and glutaric acid (0.013 g, 0.1 mmol) dissolved in 30 ml solution mixed with ethanol and water by 2:1(V/V) was heated to refluxed for 6 h and cooled to the room temperature. Single crystals suitable for *x*-ray measurements were obtained by recrystallization at room temperature.

### Refinement

The positions of H atoms, H1,H2, were found in a difference Fourier map. All the other H atoms were fixed geometrically and allowed to ride on their attached atoms, with C—H distances=0.93–0.97 Å, N—H distance=0.86Å and with *U*_iso_=1.2–1.5*U*_eq_.

### Crystal data

**Table:**

C_12_H_9_N_3_·C_5_H_8_O_4_	*Z* = 2
*M**_r_* = 327.34	*F*(000) = 344
Triclinic, *P*1	*D*_x_ = 1.390 Mg m^−^^3^
Hall symbol: -p 1	Mo *K*α radiation, λ = 0.71073 Å
*a* = 7.4384 (15) Å	Cell parameters from 25 reflections
*b* = 8.9911 (18) Å	θ = 4–14°
*c* = 11.868 (2) Å	µ = 0.10 mm^−^^1^
α = 86.67 (3)°	*T* = 293 K
β = 81.66 (3)°	Block, colorless
γ = 85.57 (3)°	0.20 × 0.17 × 0.15 mm
*V* = 782.1 (3) Å^3^	

### Data collection

**Table:**

Enraf–Nonius CAD-4 diffractometer	*R*_int_ = 0.024
Radiation source: fine-focus sealed tube	θ_max_ = 25.0°, θ_min_ = 3.1°
graphite	*h* = −7→7
ω scans	*k* = −10→10
6041 measured reflections	*l* = −14→13
2664 independent reflections	3 standard reflections every 100 reflections
1657 reflections with *I* > 2σ(*I*)	intensity decay: none

### Refinement

**Table:**

Refinement on *F*^2^	Secondary atom site location: difference Fourier map
Least-squares matrix: full	Hydrogen site location: inferred from neighbouring sites
*R*[*F*^2^ > 2σ(*F*^2^)] = 0.040	H atoms treated by a mixture of independent and constrained refinement
*wR*(*F*^2^) = 0.149	*w* = 1/[σ^2^(*F*_o_^2^) + (0.0825*P*)^2^ + 0.020*P*] where *P* = (*F*_o_^2^ + 2*F*_c_^2^)/3
*S* = 1.11	(Δ/σ)_max_ < 0.001
2664 reflections	Δρ_max_ = 0.35 e Å^−^^3^
226 parameters	Δρ_min_ = −0.32 e Å^−^^3^
1 restraint	Extinction correction: *SHELXL97* (Sheldrick, 2008), Fc^*^=kFc[1+0.001xFc^2^λ^3^/sin(2θ)]^-1/4^
Primary atom site location: structure-invariant direct methods	Extinction coefficient: 0.018 (5)

### Special details

**Table:**

Geometry. All e.s.d.'s (except the e.s.d. in the dihedral angle between two l.s. planes) are estimated using the full covariance matrix. The cell e.s.d.'s are taken into account individually in the estimation of e.s.d.'s in distances, angles and torsion angles; correlations between e.s.d.'s in cell parameters are only used when they are defined by crystal symmetry. An approximate (isotropic) treatment of cell e.s.d.'s is used for estimating e.s.d.'s involving l.s. planes.
Refinement. Refinement of *F*^2^ against ALL reflections. The weighted *R*-factor *wR* and goodness of fit *S* are based on *F*^2^, conventional *R*-factors *R* are based on *F*, with *F* set to zero for negative *F*^2^. The threshold expression of *F*^2^ > σ(*F*^2^) is used only for calculating *R*-factors(gt) *etc*. and is not relevant to the choice of reflections for refinement. *R*-factors based on *F*^2^ are statistically about twice as large as those based on *F*, and *R*- factors based on ALL data will be even larger.

### Fractional atomic coordinates and isotropic or equivalent isotropic displacement parameters (Å^2^)

**Table:**

	*x*	*y*	*z*	*U*_iso_*/*U*_eq_	
N1	0.2659 (2)	0.5349 (2)	0.58023 (17)	0.0435 (5)	
H1A	0.2940	0.6038	0.6206	0.052*	
N2	0.2164 (2)	0.4197 (2)	0.42719 (17)	0.0432 (5)	
N3	0.4250 (3)	0.9193 (2)	0.24990 (19)	0.0526 (6)	
C1	0.1814 (3)	0.3243 (2)	0.5230 (2)	0.0403 (6)	
C2	0.1261 (3)	0.1787 (3)	0.5328 (2)	0.0478 (6)	
H2B	0.1037	0.1309	0.4693	0.057*	
C3	0.1057 (3)	0.1083 (3)	0.6392 (2)	0.0515 (7)	
H3B	0.0690	0.0112	0.6477	0.062*	
C4	0.1387 (3)	0.1791 (3)	0.7345 (2)	0.0540 (7)	
H4A	0.1251	0.1275	0.8051	0.065*	
C5	0.1912 (3)	0.3235 (3)	0.7273 (2)	0.0508 (7)	
H5A	0.2117	0.3707	0.7915	0.061*	
C6	0.2117 (3)	0.3952 (3)	0.6200 (2)	0.0408 (6)	
C7	0.2664 (3)	0.5429 (2)	0.4655 (2)	0.0405 (6)	
C8	0.3189 (3)	0.6750 (3)	0.3922 (2)	0.0416 (6)	
C9	0.3303 (3)	0.6703 (3)	0.2746 (2)	0.0502 (7)	
H9A	0.3025	0.5851	0.2416	0.060*	
C10	0.3835 (3)	0.7942 (3)	0.2073 (2)	0.0563 (7)	
H10A	0.3908	0.7903	0.1286	0.068*	
C11	0.4129 (3)	0.9229 (3)	0.3626 (2)	0.0520 (7)	
H11A	0.4408	1.0097	0.3934	0.062*	
C12	0.3614 (3)	0.8050 (3)	0.4361 (2)	0.0488 (6)	
H12A	0.3553	0.8125	0.5144	0.059*	
O1	−0.3978 (3)	0.1206 (2)	0.12019 (17)	0.0654 (6)	
O2	−0.3751 (2)	0.2383 (2)	0.27704 (16)	0.0599 (5)	
O3	0.2390 (4)	0.3965 (3)	0.1101 (2)	0.1041 (9)	
O4	0.1264 (3)	0.2629 (2)	0.26029 (18)	0.0776 (7)	
C13	−0.3421 (3)	0.2273 (3)	0.1748 (2)	0.0490 (6)	
C14	−0.2312 (4)	0.3340 (3)	0.0967 (2)	0.0618 (8)	
H14A	−0.1852	0.4040	0.1429	0.074*	
H14B	−0.3110	0.3908	0.0497	0.074*	
C15	−0.0728 (4)	0.2637 (3)	0.0197 (2)	0.0597 (7)	
H15A	−0.0195	0.3404	−0.0327	0.072*	
H15B	−0.1176	0.1914	−0.0252	0.072*	
C16	0.0758 (4)	0.1854 (3)	0.0833 (2)	0.0619 (8)	
H16A	0.0244	0.1063	0.1340	0.074*	
H16B	0.1714	0.1405	0.0288	0.074*	
C17	0.1557 (4)	0.2930 (3)	0.1513 (3)	0.0601 (8)	
H1	0.173 (5)	0.338 (4)	0.308 (3)	0.123 (13)*	
H2	−0.456 (4)	0.058 (4)	0.167 (3)	0.133 (15)*	

### Atomic displacement parameters (Å^2^)

**Table:**

	*U*^11^	*U*^22^	*U*^33^	*U*^12^	*U*^13^	*U*^23^
N1	0.0502 (11)	0.0392 (11)	0.0437 (13)	−0.0103 (8)	−0.0095 (9)	−0.0083 (9)
N2	0.0464 (11)	0.0400 (12)	0.0465 (13)	−0.0084 (9)	−0.0127 (9)	−0.0063 (10)
N3	0.0573 (12)	0.0472 (13)	0.0568 (15)	−0.0157 (10)	−0.0133 (10)	−0.0024 (11)
C1	0.0380 (12)	0.0384 (13)	0.0466 (15)	−0.0042 (9)	−0.0102 (10)	−0.0064 (11)
C2	0.0524 (14)	0.0402 (14)	0.0547 (17)	−0.0087 (11)	−0.0144 (11)	−0.0120 (12)
C3	0.0589 (15)	0.0391 (14)	0.0592 (18)	−0.0121 (11)	−0.0122 (12)	−0.0039 (13)
C4	0.0646 (16)	0.0458 (15)	0.0515 (17)	−0.0079 (12)	−0.0054 (13)	−0.0029 (13)
C5	0.0653 (16)	0.0461 (15)	0.0427 (16)	−0.0088 (12)	−0.0079 (12)	−0.0089 (12)
C6	0.0403 (12)	0.0364 (13)	0.0472 (15)	−0.0047 (9)	−0.0075 (10)	−0.0084 (11)
C7	0.0375 (12)	0.0408 (13)	0.0454 (15)	−0.0066 (10)	−0.0098 (10)	−0.0061 (11)
C8	0.0387 (12)	0.0406 (13)	0.0475 (15)	−0.0064 (9)	−0.0089 (10)	−0.0071 (11)
C9	0.0582 (15)	0.0474 (15)	0.0499 (16)	−0.0168 (11)	−0.0160 (12)	−0.0054 (12)
C10	0.0678 (17)	0.0572 (17)	0.0500 (17)	−0.0221 (13)	−0.0207 (13)	0.0012 (13)
C11	0.0566 (15)	0.0448 (15)	0.0569 (18)	−0.0151 (11)	−0.0064 (12)	−0.0102 (13)
C12	0.0523 (14)	0.0485 (15)	0.0470 (16)	−0.0128 (11)	−0.0049 (11)	−0.0082 (12)
O1	0.0996 (15)	0.0540 (12)	0.0469 (12)	−0.0322 (11)	−0.0076 (10)	−0.0088 (10)
O2	0.0784 (12)	0.0601 (12)	0.0441 (12)	−0.0230 (9)	−0.0063 (9)	−0.0081 (9)
O3	0.170 (2)	0.0867 (17)	0.0689 (16)	−0.0736 (17)	−0.0346 (15)	0.0149 (13)
O4	0.1193 (18)	0.0732 (15)	0.0487 (14)	−0.0464 (13)	−0.0185 (12)	−0.0038 (11)
C13	0.0623 (15)	0.0415 (14)	0.0453 (17)	−0.0102 (11)	−0.0097 (12)	−0.0044 (12)
C14	0.090 (2)	0.0469 (16)	0.0497 (17)	−0.0231 (14)	−0.0054 (15)	−0.0005 (13)
C15	0.0806 (18)	0.0623 (18)	0.0390 (16)	−0.0253 (14)	−0.0061 (13)	−0.0068 (13)
C16	0.088 (2)	0.0522 (17)	0.0492 (18)	−0.0181 (14)	−0.0124 (14)	−0.0107 (14)
C17	0.0833 (19)	0.0498 (17)	0.0520 (19)	−0.0205 (14)	−0.0195 (14)	0.0043 (14)

### Geometric parameters (Å, °)

**Table:**

N1—C7	1.359 (3)	C9—H9A	0.9300
N1—C6	1.385 (3)	C10—H10A	0.9300
N1—H1A	0.8600	C11—C12	1.375 (3)
N2—C7	1.320 (3)	C11—H11A	0.9300
N2—C1	1.390 (3)	C12—H12A	0.9300
N3—C11	1.330 (3)	O1—C13	1.313 (3)
N3—C10	1.334 (3)	O1—H2	0.866 (10)
C1—C2	1.396 (3)	O2—C13	1.211 (3)
C1—C6	1.402 (3)	O3—C17	1.199 (3)
C2—C3	1.373 (3)	O4—C17	1.296 (3)
C2—H2B	0.9300	O4—H1	1.02 (4)
C3—C4	1.390 (4)	C13—C14	1.503 (3)
C3—H3B	0.9300	C14—C15	1.504 (4)
C4—C5	1.379 (4)	C14—H14A	0.9700
C4—H4A	0.9300	C14—H14B	0.9700
C5—C6	1.387 (4)	C15—C16	1.532 (4)
C5—H5A	0.9300	C15—H15A	0.9700
C7—C8	1.475 (3)	C15—H15B	0.9700
C8—C12	1.383 (3)	C16—C17	1.503 (4)
C8—C9	1.389 (4)	C16—H16A	0.9700
C9—C10	1.381 (3)	C16—H16B	0.9700
			
C7—N1—C6	107.18 (19)	C9—C10—H10A	118.5
C7—N1—H1A	126.4	N3—C11—C12	123.4 (2)
C6—N1—H1A	126.4	N3—C11—H11A	118.3
C7—N2—C1	105.23 (19)	C12—C11—H11A	118.3
C11—N3—C10	117.5 (2)	C11—C12—C8	119.2 (2)
N2—C1—C2	130.2 (2)	C11—C12—H12A	120.4
N2—C1—C6	109.62 (19)	C8—C12—H12A	120.4
C2—C1—C6	120.2 (2)	C13—O1—H2	111 (3)
C3—C2—C1	117.8 (2)	C17—O4—H1	114 (2)
C3—C2—H2B	121.1	O2—C13—O1	123.9 (2)
C1—C2—H2B	121.1	O2—C13—C14	123.5 (2)
C2—C3—C4	121.4 (2)	O1—C13—C14	112.6 (2)
C2—C3—H3B	119.3	C13—C14—C15	115.5 (2)
C4—C3—H3B	119.3	C13—C14—H14A	108.4
C5—C4—C3	121.9 (2)	C15—C14—H14A	108.4
C5—C4—H4A	119.0	C13—C14—H14B	108.4
C3—C4—H4A	119.0	C15—C14—H14B	108.4
C4—C5—C6	116.9 (2)	H14A—C14—H14B	107.5
C4—C5—H5A	121.6	C14—C15—C16	113.8 (2)
C6—C5—H5A	121.6	C14—C15—H15A	108.8
N1—C6—C5	133.1 (2)	C16—C15—H15A	108.8
N1—C6—C1	105.2 (2)	C14—C15—H15B	108.8
C5—C6—C1	121.7 (2)	C16—C15—H15B	108.8
N2—C7—N1	112.81 (19)	H15A—C15—H15B	107.7
N2—C7—C8	124.0 (2)	C17—C16—C15	111.4 (2)
N1—C7—C8	123.2 (2)	C17—C16—H16A	109.3
C12—C8—C9	117.8 (2)	C15—C16—H16A	109.3
C12—C8—C7	122.3 (2)	C17—C16—H16B	109.3
C9—C8—C7	119.9 (2)	C15—C16—H16B	109.3
C10—C9—C8	118.9 (2)	H16A—C16—H16B	108.0
C10—C9—H9A	120.5	O3—C17—O4	122.6 (3)
C8—C9—H9A	120.5	O3—C17—C16	124.1 (3)
N3—C10—C9	123.1 (3)	O4—C17—C16	113.3 (2)
N3—C10—H10A	118.5		
			
C7—N2—C1—C2	−179.2 (2)	N2—C7—C8—C12	176.6 (2)
C7—N2—C1—C6	0.2 (2)	N1—C7—C8—C12	−4.0 (3)
N2—C1—C2—C3	178.4 (2)	N2—C7—C8—C9	−4.7 (3)
C6—C1—C2—C3	−1.0 (3)	N1—C7—C8—C9	174.8 (2)
C1—C2—C3—C4	0.0 (4)	C12—C8—C9—C10	0.2 (4)
C2—C3—C4—C5	0.8 (4)	C7—C8—C9—C10	−178.6 (2)
C3—C4—C5—C6	−0.7 (4)	C11—N3—C10—C9	−0.3 (4)
C7—N1—C6—C5	178.0 (2)	C8—C9—C10—N3	0.0 (4)
C7—N1—C6—C1	0.0 (2)	C10—N3—C11—C12	0.4 (4)
C4—C5—C6—N1	−178.0 (2)	N3—C11—C12—C8	−0.2 (4)
C4—C5—C6—C1	−0.2 (3)	C9—C8—C12—C11	−0.1 (3)
N2—C1—C6—N1	−0.1 (2)	C7—C8—C12—C11	178.7 (2)
C2—C1—C6—N1	179.4 (2)	O2—C13—C14—C15	126.6 (3)
N2—C1—C6—C5	−178.4 (2)	O1—C13—C14—C15	−52.9 (3)
C2—C1—C6—C5	1.1 (3)	C13—C14—C15—C16	−64.9 (3)
C1—N2—C7—N1	−0.2 (2)	C14—C15—C16—C17	−60.6 (3)
C1—N2—C7—C8	179.28 (19)	C15—C16—C17—O3	−64.2 (4)
C6—N1—C7—N2	0.2 (2)	C15—C16—C17—O4	116.1 (3)
C6—N1—C7—C8	−179.33 (19)		

### Hydrogen-bond geometry (Å, °)

**Table:**

*D*—H···*A*	*D*—H	H···*A*	*D*···*A*	*D*—H···*A*
N1—H1A···O2^i^	0.86	2.10	2.957 (3)	176.
O1—H2···N3^ii^	0.87 (1)	1.75 (1)	2.615 (3)	173 (4)
O4—H1···N2	1.02 (4)	1.71 (4)	2.686 (3)	158 (3)

Symmetry codes: (i) −*x*, −*y*+1, −*z*+1; (ii) *x*−1, *y*−1, *z*.
